# Cost-Effective, Implant-Free, All-Suture Modified Subpectoral Biceps Tenodesis Technique

**DOI:** 10.1016/j.eats.2023.11.001

**Published:** 2024-01-01

**Authors:** Anna Jacques, Charles A. Slater, James Ross

**Affiliations:** a Department of Orthopedic Surgery, Broward Health Medical Center, Fort Lauderdale, Florida, U.S.A.; b Baptist Health Orthopedic Care, Deerfield Beach, Florida, U.S.A.

## Abstract

Tendinopathy and partial tears of the long head of the biceps tendon are a common cause of anterior shoulder pain and are often associated with many other shoulder pathologies. Multiple open and arthroscopic tenodesis techniques exist in the literature, with varying locations along the proximal humerus and a multitude of different implants. This article describes a cost-effective, implant-free, subpectoral biceps tenodesis technique that can be used as open or in conjunction with arthroscopy. Our technique differs from other tunnel techniques in its modified docking configuration into the intraosseous canal, decreasing stresses at the bone-tendon interface. With the cost amounting to 5% to 10% of techniques using implants, this is a cost-saving and reliable option for tenodesis.

A plethora of literature exists to date regarding the treatment of tendinopathy of the long head of the biceps brachii tendon (LHBT). When nonsurgical measures fail to resolve symptoms, surgical treatment may involve either a biceps tenotomy or a biceps tenodesis.^1^ The incidence of cosmetic deformity and fatigue discomfort has been proven to be less in the tenodesis cohorts when compared to a tenotomy.^1^, ^2^, ^3^

The biceps tenodesis technique has been described at varying locations along the course of the long head of the biceps tendon. These locations which have previously been described have included intra-articular, suprapectoral, and subpectoral. Both open and arthroscopic techniques have been described for these varying locations.^2^ There is a growing concern for persistent symptoms when the tenodesis has been performed in the bicipital groove, thought to be related to persistent tenosynovitis.^3^ This concern has led to the development of a subpectoral biceps tenodesis to eliminate the chance of bicipital groove pain. Tenodesis can be achieved using implants for the varying techniques that range in price from $500 to more than $1,000. We describe an intraosseous, cost-effective, clinically reproducible, subpectoral tenodesis technique that costs approximately 5% to 10% (Table 1) of other described techniques and uses standard readily available surgical equipment while eliminating additional stress on the LHBT.

**Table 1:** Implant Costs

Implant	Cost
Our technique: 18 gauge spinal needle, Ultrabraid suture,∗ PDS suture†	$56
2.8 mm Q-Fix All Suture Anchor∗	$517
2 × 1.8mm Q-Fix All Suture Anchor∗	$535
Biosure Interference Screw∗	$525
Tenodesis Screw‡	$550
FiberTak Anchor‡	$550
Cortical Button‡	$600
Iconix§	$347
Interference Screw§	$264
G-Lok§	$236
ProCinch§	$481

The implant costs for varying biceps tenodesis technique implants were obtained from a local surgery center (Coconut Creek, FL) and hospital (Boca Raton, FL) and averaged to compare the costs of the various commonly used implants. Our novel intraosseous docking, subpectoral biceps tenodesis technique costs $56, which is approximately 5% to 10% of the cost of techniques using implants.

∗ Smith & Nephew, London, UK.

† Ethicon, Raritan, NJ.

‡ Arthrex, Inc. Naples, FL.

§ Stryker, Kalamazoo, MI, USA.

## Surgical Technique

Patient is placed in beach chair position, with the forearm held by a dynamic limb positioner. Shoulder arthroscopy is initially performed, with relevant surgical procedures implemented depending on the patient’s pathology in the glenohumeral joint and subacromial space. During the arthroscopic examination of the shoulder, a tenotomy of the long head of the biceps is performed at the level of the tendon-labrum junction with an arthroscopic scissor. Gentle debridement of the long head of the biceps may also be performed with an arthroscopic shaver to ease the passing of the tendon through the bicipital groove for later tenodesis.

**Fig 1:**
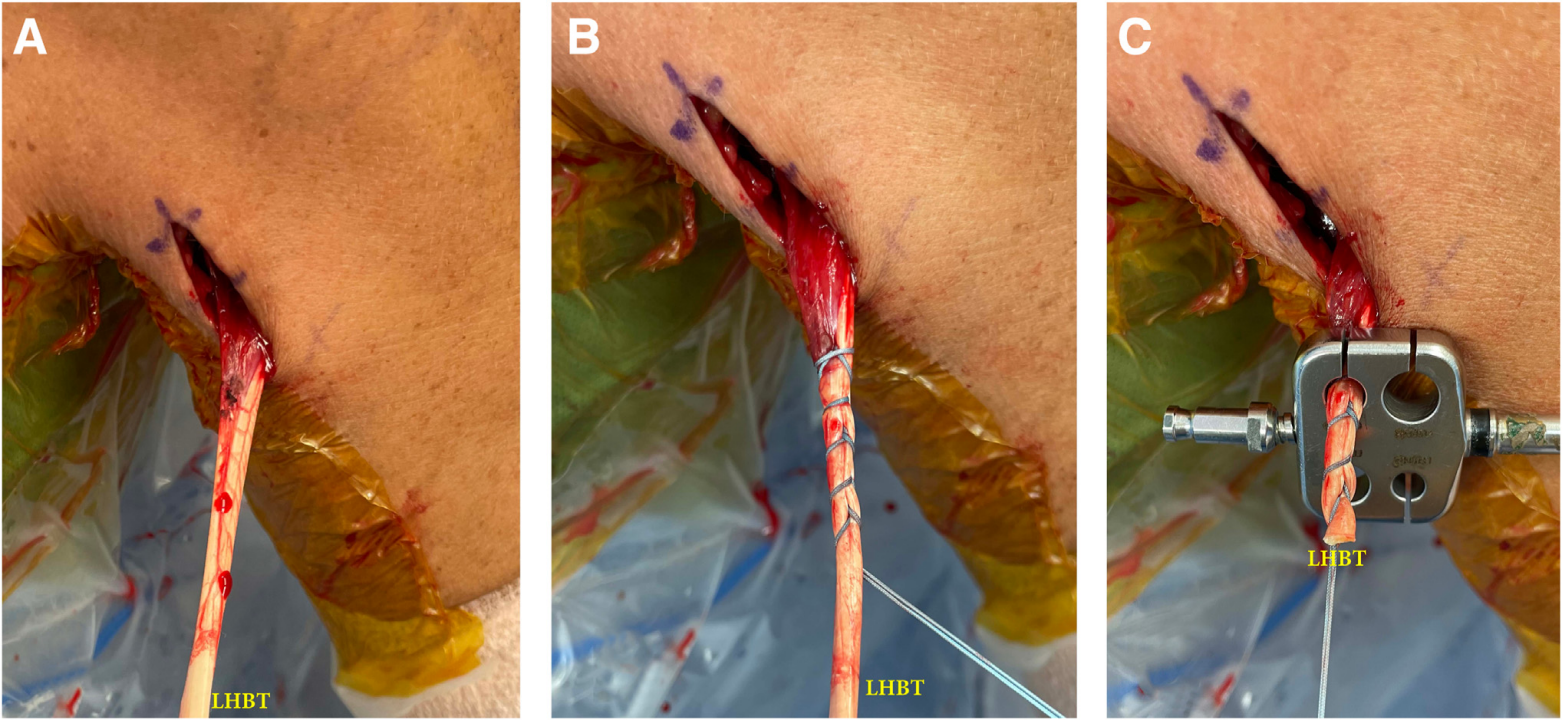
Direct view of the left proximal arm subpectoral mini-open incision made at the inferior border of the pectoralis major with the patient in the beach chair position. (A) The long head of biceps tendon (LHBT) is retrieved out of the incision. (B) A 2.0 looped Ultrabraid suture (Smith & Nephew, London, UK) used to whipstitch the tendon beginning at the myotendinous junction and ending 30 mm proximally. (C) Proximal tendon is sharply amputated, and the remaining tendon is then sized with a standard graft sizer.

**Fig 2:**
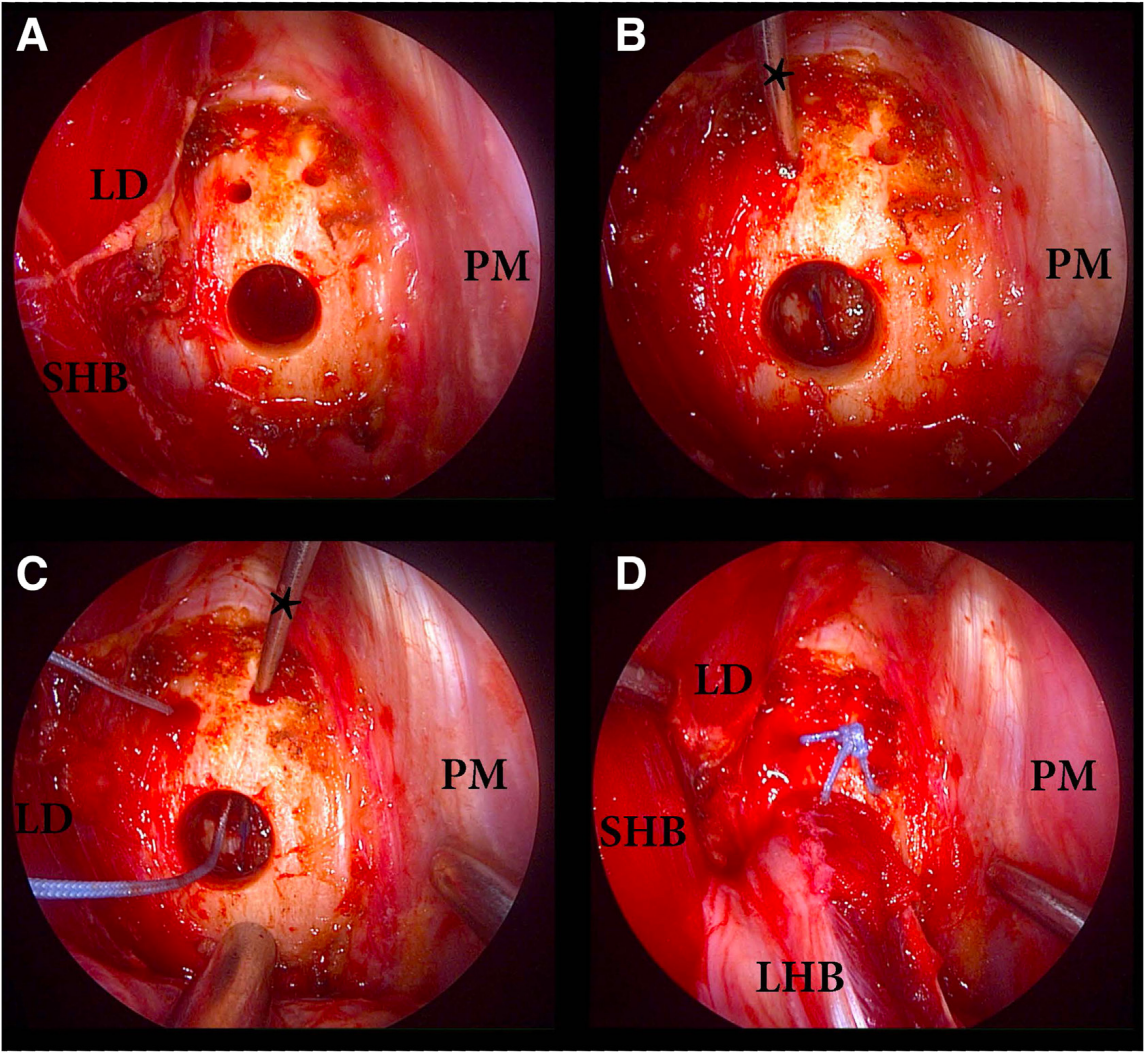
Deep view of the left anterior proximal arm subpectoral mini-open incision made at the inferior border of the pectoralis major (PM) with the patient in the beach chair position. The PM muscle is retracted superiorly (top of view). The short head of the biceps (SHB) muscle and latissimus dorsi (LD) insertion retracted medial (left side of view), and PM insertion retracted lateral (right side of view). Periosteum of the humerus has been cleared with electrocautery. (A) Configuration of drill holes. (B) A spinal needle(∗) is used to pass a 1.0 polydioxanone (PDS) suture (Ethicon, Bridgewater, NJ) through the docking hole. (C) A 1.0 PDS suture (Ethicon) is used to shuttle a 2.0 Ultrabraid suture (Smith & Nephew, London, UK) into 2 mm holes in retrograde fashion through the docking hole. (D) The long head of the biceps (LHB) is docked into a bone tunnel with 2.0 Ultrabraid sutures secured over the bone bridge.

Once the arthroscopic tenotomy is complete, the upper extremity is then placed in a slightly abducted and externally rotated position allowing access to the biceps groove (Video 1). The inferior aspect of the pectoralis major (PM) tendon insertion is palpated and marked. An approximately 3 cm longitudinal skin incision is made beginning 1 cm proximal to the inferior border of the pectoralis major tendon and continued 2 cm distally. Dissection with electrocautery and Metzenbaum scissors is performed to the level of the biceps brachii fascia. The inferior border of the pectoralis major tendon is identified, dissected off the brachial fascia, and retracted superiorly. The long head of the biceps tendon is identified within the bicipital groove, just medial to the pectoralis major tendon insertion on the humerus. An allis clamp is then used to deliver the tendon out of the surgical incision. A 2.0 looped Ultrabraid suture (Smith & Nephew, London, UK) with a curved needle is used to whipstitch the tendon beginning at the myotendinous junction and ending 30 mm proximally. Careful consideration is made to change the angle of each needle pass through the tendon to make a multiplanar perpendicular whipstitch because this has shown to be stronger than a standard whipstitch.^2^ The remaining tendon is sharply amputated with a 15-blade scalpel. The tendon is then sized with a standard graft sizer (Fig 1). The periosteum of the bicipital groove is then cleared with electrocautery, with care to avoid releasing the pectoralis major or latissimus dorsi tendon insertions. A 2.4 mm anterior cruciate ligament guide pin is then drilled unicortically in the planned area of the tendon socket, approximately 3 cm proximal from the inferior border of the PM. See technical pearls for further exposure and tendon preparation tips (Table 2). A cannulated anterior cruciate ligament reamer of the respective tendon diameter size is unicortically drilled over the guide pin. Next, a 2.0 mm drill is used to drill 2 unicortical holes approximately 5 mm superolateral and superomedial to the previously drilled unicortical socket leaving an approximate 5 mm bony bridge between the two holes (Fig 2A).

**Table 2:** Technical Pearls

Incision is made over the inferior PM border.
Avoid any medial retractor placement. Use palpation to confirm location over the anterior aspect of the humerus. The short head of the biceps muscle belly leads to the LHBT just lateral.
Use army navy retractor to lift PM superiorly.
Use ruler to measure 2 cm from the PM superior border or 3 cm from the inferior PM border.^11^
Begin whipstitch at musculotendinous junction and continue for 30 mm proximal. Change the angle of each needle pass.^4^
Make sure tendon easily passes within the sized diameter docking hole.

PM, pectoralis major; LHBT, long head biceps tendon.

An 18 gauge spinal needle is then bent approximately 60° to 70° at its tip and loaded with a 1.0 polydioxanone (PDS) suture (Ethicon, Bridgewater, NJ, USA). The spinal needle is then inserted into one of the 2.0 mm suture drill holes and into the humeral canal, with the bent portion of the needle aimed toward the tendon docking tunnel. The PDS suture is then advanced through the spinal needle and thus through the humeral canal toward the docking tunnel (Fig 2B). Once the PDS suture is visualized at the base of the docking tunnel, a thin-tipped tonsil is used to retrieve the suture out of the docking socket. This suture is then used as a shuttling suture by tying a loop at the free end and passing one of the Ultrabraid graft suture limbs through the loop. The PDS/Ultrabraid suture is then passed retrograde into the docking tunnel and out of the 2.0 mm suture tunnel. This process is repeated for the other limb of the Ultrabraid suture through the remaining free 2.0 mm drill hole (Fig 2C). Both limbs of the Ultrabraid suture are then tensioned, allowing for docking of the long head of the biceps tendon into the tunnel. The Ultrabraid suture is then hand-tied over the bony bridge in a standard fashion (Fig 2D). The incision is then copiously irrigated and a layered closure is performed.

Postoperative protocols vary depending on the concomitant pathology and other surgical procedures performed at the time of the biceps tenodesis. If an isolated biceps tenodesis is performed with no other surgical procedures requiring specific postoperative immobilization, the extremity is placed into a standard sling with no active elbow flexion or supination for 6 weeks.

## Discussion

**Fig 3:**
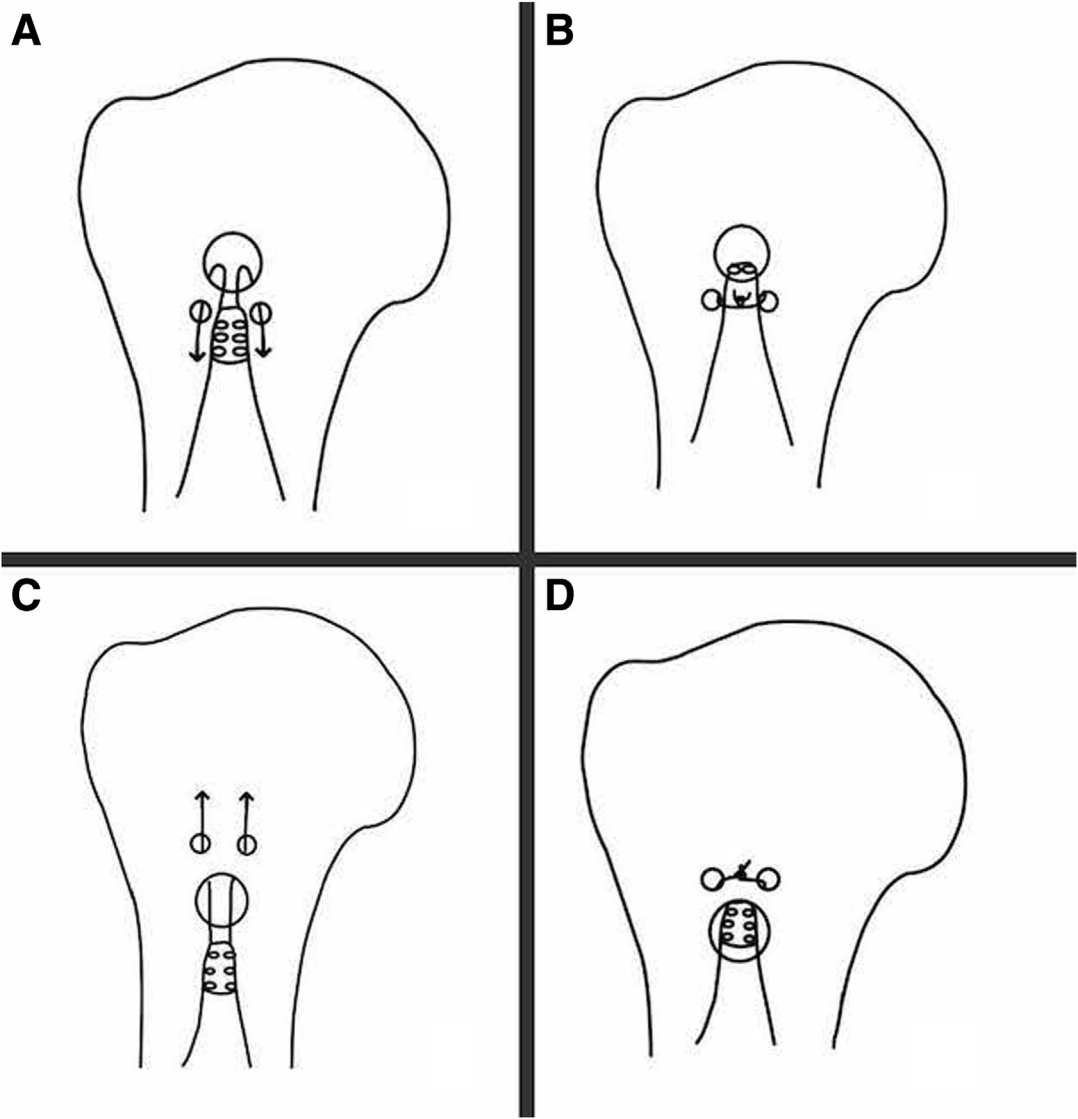
A right proximal humerus with the original intraosseous biceps tenodesis tunnel technique (A, B) and our described modified technique for comparison (C, D). The original intraosseous docking technique tunnels the long head biceps tendon into the proximal larger hole, securing the tendon with a 180° turn and then tying the sutures over the tendon at the level of the 2 smaller-diameter holes distally^6^ (A, B). Our modification of the technique consists of placing the suture drill holes proximal to the docking tunnel, eliminating the 180° turn of the tendon around the cortical bone and instead allowing the tendon to rest underneath the cortical bone with sutures tied over a proximal bone bridge (C, D).

Intraosseous implant-free biceps tenodesis was a technique originally described by Post and Benca^5^ in 1989, which was later popularized by Snyder as the tunnel technique. The technique consisted of drilling a larger-diameter docking hole with 2 corresponding smaller holes distally. As the LHBT was docked into the larger hole, it took a 180° turn, and the sutures were tied over the tendon at the level of the 2 smaller-diameter holes distally^6^ (Figs 3A, 3B). This technique has shown favorable results in multiple biomechanical studies.^6^^,^^7^ In one biomechanical study, Ozalay et al.^6^ demonstrated the tunnel technique to have a mean maximum failure load of 229 N, falling just short of the interference screw technique which failed at 243 N. Sampatacos et al.^7^ compared an arthroscopic biceps intraosseous tenodesis with interference screws and found that it failed at nearly 2.5 times the load compared to interference screws during maximal load to failure testing. One aspect of the original intraosseous technique we believe to be disadvantageous is the 180° turn that the tendon must undergo as it passes around the cortical bone (Fig 3B). We believe this may cause significant tendon-bone motion and be a potential for failure.^7^^,^^8^ This concept led to our modification of the technique by placing the suture drill holes proximal to the docking tunnel, eliminating the 180° turn of the tendon around the cortical bone and instead allowing the tendon to rest underneath the cortical bone with decreased interface stress (Figs 3C, 3D).

There are several additional advantages to our modified intraosseous docking technique (Table 3). First, removing the biceps tendon from the bicipital groove decreases the rates of persistent “bicipital groove pain” and revision tenodesis surgery.^7^ Second, the mini-open subpectoral approach has a much lower learning curve and removes the potential complications of prolonged arthroscopic procedures around the shoulder. Although we do recognize that there are risks to the open procedure given its proximity to the axilla, when compared to arthroscopic suprapectoral tenodesis, the increased rate of wound complications (1.0% vs 0.0%) and nerve injury (0.7% vs 0.0%) are low.^9^^,^^10^ Furthermore, the location of our docking hole is strategically located to provide an optimal intraosseous LHBT length. Our tenodesis location of 3 cm proximal from the inferior border of the PM places our tenodesis location at the cranial half of PM, in an area of increased tendon to muscle ratio compared to the inferior edge of the PM.^11^ This results in increased tendon length within the humeral canal, which promotes tendonous healing within the intraosseous cortex allowing for a biological increase in strength with healing.^7^ We do recognize that there is a small inherent risk with every subpectoral tenodesis technique of spiral fracture and decreased torsional strength of the humerus which needs to be considered.^12^ Furthermore, the technique is cost effective, making it both an economically and biomechanically sound option.

**Table 3:** Technique Specific Advantages and Disadvantages

Advantages
Elimination of proximal bicipital groove anterior shoulder pain
Cost saving
Does not rely on a specific manufacturer or representative
Decreased risk of adverse arthroscopic events seen with lengthy arthroscopic techniques
Tenodesis placed in location of maximal tendon to muscle ratio^11^
No known clinical failures over 3 years
Disadvantages
No biomechanical data for modified tunnel technique
Risk of spiral fracture due to decreased torque and rotational force to failure^12^
0.7% reported risk of neurovascular injury with subpectoral tenodesis techniques^9^^,^^10^
1.0% reported risk of wound complications for subpectoral tenodesis techniques^9^^,^^10^

## Disclosures

The authors declare the following financial interests/personal relationships which may be considered as potential competing interests: J.R. is a paid consultant for Smith & Nephew.

## Supplementary Data

ICMJE author disclosure forms

Video 1 Cost-effective, implant-free, all-suture modified subpectoral biceps tenodesis technique video. With the patient in the beach chair position, the left shoulder and proximal arm are prepped and draped in standard orthopaedic fashion.
