# Supplementary material for: Cost-Effective, Implant-Free, All-Suture Modified Subpectoral Biceps Tenodesis Technique
Source: Arthrosc Tech. 2024 Jan 1;13(3):102873. doi: 10.1016/j.eats.2023.11.001 (PMC10995638; doi:10.1016/j.eats.2023.11.001)
Supplement: ICMJE author disclosure forms [file mmc1.docx]

**Declaration of interests**
 
☐ The authors declare that they have no known competing financial interests or personal relationships that could have appeared to influence the work reported in this paper.
 
☒ The authors declare the following financial interests/personal relationships which may be considered as potential competing interests:

| James Ross reports a relationship with Smith and Nephew Inc that includes: consulting or advisory. |
| --- |

**Declaration of interests**
 
☒ The authors declare that they have no known competing financial interests or personal relationships that could have appeared to influence the work reported in this paper.
 
☐ The authors declare the following financial interests/personal relationships which may be considered as potential competing interests:

**Declaration of interests**
 
☒ The authors declare that they have no known competing financial interests or personal relationships that could have appeared to influence the work reported in this paper.
 
☐ The authors declare the following financial interests/personal relationships which may be considered as potential competing interests:
